# A comparison of the performance and clinical utility of mid-molecular and C-terminal parathyroid hormone assays

**DOI:** 10.1155/S1463924687000348

**Published:** 1987

**Authors:** Rosemarie Freaney, Martin Quinn, Francis P. Muldowney

**Affiliations:** 1 Department of Medicine and Renal Disease St. Vincents Hospital Dublin 4 Ireland; 2 Department of Medicine University College Dublin 4 Ireland


Journal of Automatic Chemistry/*Journal of Clinical Laboratory Automation*, Vol. 9, No. 4 (October–December 1987), pp. 174–178

### A comparison of the performance and clinical utility of mid-molecular and C-terminal parathyroid hormone assays

**Rosemarie Freaney, Martin Quinn and Francis P. Muldowney**

*Department of Medicine and Renal Disease, St. Vincents Hospital, and Department of Medicine, University College, Dublin 4, Eire*

*Two methods for parathyroid hormone assay in serum using commercially available reagents are compared. The Omega Radioimmunoassay uses an antiserum with recognition for the mid-molecular region of parathyroid hormone (amino acid 53–68). The Dac cell method measures both intact hormone and C-terminal fragments.*

*Both assays can be completed within 24 h and offer good diagnostic sensitivity for primary hyperparathyroidism (PHPT). The C-terminal assay showed increased values in 85% of proven PHPT patients compared to 96% for the mid-molecule PTH method.*

*The C-terminal assay found 73% of hypoparathyroid patients to have undetectable PTH levels in serum, but 54% of values in healthy subjects were undetectable by this method.*

*The mid-molecule assay was clearly better in distinguishing hypoparathyroid patients from healthy subjects, as serum PTH was undetectable in 73% of hypoparathyroid patients and detectable in 92% of healthy subjects.*

### Introduction

Radioimmunoassay represented a major methodological advance in endocrinology. However, the immunoheterogeneity of some hormones resulted in varying diagnostic sensitivities and specificities and limited their clinical utility. Parathyroid hormone (PTH) was one such hormone, being rapidly metabolized in liver and kidney [1] to yield aminoterminal (amino acid 1–34) and carboxyl terminal (aa 35–85) and other polypeptides. Armitage [2] in a recent review of parathyroid assay performance listed reported studies showing that C-terminal and mid-molecular assays provided clearer discrimination than N-terminal assays between normal and abnormal parathyroid function. The N-terminal assays reflect better acute changes in parathyroid function [2 and 3].

In this report two commercial assays – one with C-terminal and whole hormone specificity (Dac cell) and one with recognition for the mid-molecular region of PTH (Omega assay) are compared, and their clinical utility are discussed with particular reference to the prevalence of primary hyperparathyroidism in various population groups.

### Material and methods

The commercially available PTH kits were:

(1) Dac cell PTH assay RD51 supplied by Wellcome Diagnostics, Dartford DA15 AH, UK.
(2) Omega PTH kit – Cambridge Medical Diagnostics available from Biogenesis, Bucklers Way, Bournemouth, UK.

Assay details relating to the antiserum characterization and tracer used, the method of standardization, phase separation and the quality-assurance materials used are included in table 1. The quality of packaging and labelling of both kits is in accordance with IFCC recommendations [4].

The assay protocol used was that supplied by the manufacturer. Precision was assessed at two levels both within and between batch by replicate analysis of pooled human serum. Other analytical techniques used were atomic absorption spectroscopy for total calcium, ionized calcium by ion sensitive electrode Orion SS 20 calcium analyser.

### *Statistical methods*

Group means were performed with the standard deviation as an index of dispersion. Linear regression analysis was performed by the method of least squares, and the difference between means by the paired or unpaired T test where appropriate.

### Stability of PTH

Ingle *et al.* [5] have previously reported data showing the stability of C-terminal PTH fragments in serum. No significant change in PTH occurred in serum left at room temperature for 24 h, even if not centrifuged and separated, and no significant change in PTH was reported after one, two or three freeze thaw cycles.

Our stability studies with the mid-molecule PTH assay show that no marked decrease in PTH occurred when the blood was centrifuged and the separated serum left on the bench for 24 h at room temperature (17–21 °C) (see figure 1). When a small number of serum samples subjected to one freeze/thaw cycle ($N = 6$) or two freeze/thaw cycles ($N = 4$) were compared to the basal value, no marked change in PTH occurred (see figure 1).

*Table 1. C-terminal (Dac cell) and mid-molecule (Omega) PTH assay details.*

| | DAC cell assay | Omega assay |
|---|---|---|
| Characterization | Recognizes 1·84 and C-terminal PTH fragments | Recognizes 1·84 and all fragments containing 53–68 aa sequence |
| Anti-serum | Anti-bovine PTH raised in guinea-pig | Goat anti-human PTH aa seq. 53–68 |
| Tracer | Bovine PTH $I^{125}$ labelled to sp. act. of approx. 150 uCi/mg (yellow dye incorporated) | $I^{125}$ labelled tyrosylated PTH (44–68 aa seq.) |
| Standardization | Bovine PTH in human Serum – conc. up to 8 µg/l: bPTH calibrated against MRC hPTH (1–84) 79/500 | Human PTH – 1·84 0·5–15 µg/l Calibrated against WHOhPTH Reference preparation 79/500 |
| Separation | Second antibody: donkey anti-guinea-pig serum covalently linked to cellulose | Second antibody: donkey Anti-goat gamma globulin |
| Reference material/ PTH control | Freeze-dried human serum enriched with human PTH Control serum contains thiomeorsal as preservative | Processed human serum containing synthetic human PTH plus preservative |

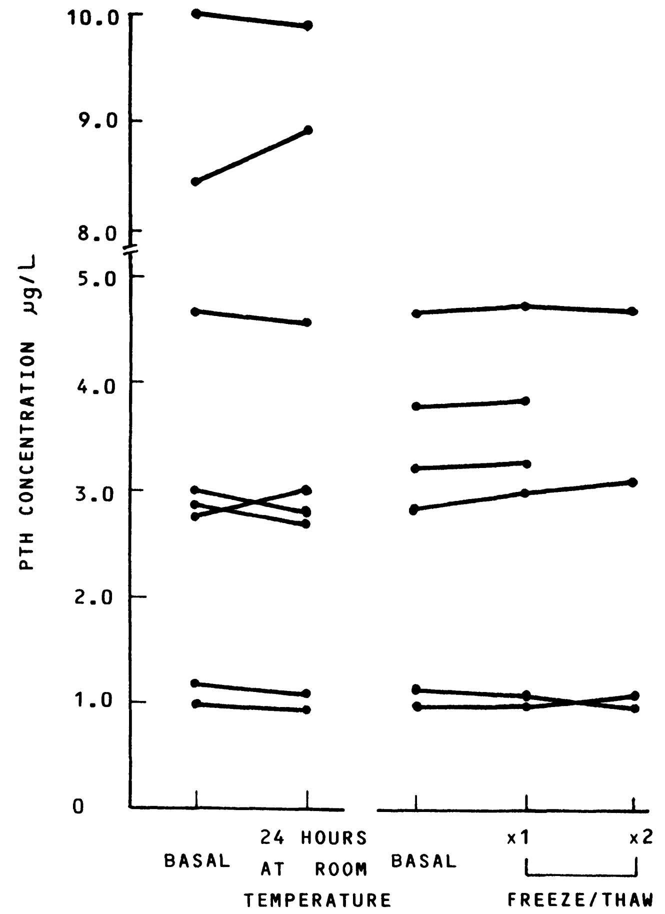

*Figure 1. PTH concentration (mid-molecule assay) in serum separated and frozen within 1 h (basal) compared to PTH concentration in serum stored at room temperature (17–21° C) for 24 h. Basal serum PTH concentrations are compared to PTH levels following one/two freeze-thaw cycles.*

### Results

The precision, both within and between batch, for both assays is shown in table 2. Between-batch precision was similar for both assays giving CV values of 10·4% and 10·6% for the C-terminal assay and 9·4% and 10·2% for the mid-molecule PTH assay. Recovery of added PTH from serum gave a mean value of 92·4% (range 90·9–93·3) for the C-terminal assay, compared to a mean recovery of 92·3% (80–103·7%) for the mid molecular PTH method ($N = 6$). A series of replicates of the zero standard ($N = 20$) were assayed and two standard deviations from the mean PTH concentration was 0·13 µg/l and 0·27 µg/l of serum for the C-terminal and mid-molecular assays respectively. This was the calculated minimum detectable amount of PTH in serum.

A reference range was established by assay of PTH in the serum of apparently healthy subjects – age group 18–62 years – $N = 41$ for the C-terminal assay and $N = 26$ for the mid-molecular assay. Using the C-terminal assay, serum PTH was detectable in 19 of 41 subjects and ranged from 0·13 µg/l to 0·40 µg/l. Log transformation of this data yielded a 95% range of 0·08–0·34 µg/l. Serum PTH was detectable in all but two of the 26 control subjects. When the mid-molecular PTH assay was performed, the mean PTH value was 0·69 ng/ml and the 95% (± 2SD) range was 0·19–1·19 µg/l. Log transformation of this data yielded a 95% range of 0·31–1·36 µg/l.

### Primary hyperparathyroidism (PHPT)

Serum PTH (C-terminal) in 20 patients with surgically proven PHPT was higher than control values in all but three cases (see figure 2). The suspected PHPT group were hypercalcaemic patients in whom the diagnosis of

*Table 2. Precision of PTH measurements using the C-terminal (Dac Cell) and mid-molecule (Omega) assays.*

| | Within batch | | | Between batch | | |
|---|---|---|---|---|---|---|
| | μg/l | N | CV % | μg/l | N | CV % |
| C-terminal assay | 0·32 | 20 | 8·8 | 0·76 | 20 | 10·4 |
| | 0·46 | 20 | 10·3 | 2·25 | 20 | 10·6 |
| Mid-molecular assay | 0·90 | 20 | 12·8 | 1·04 | 20 | 9·4 |
| | 9·50 | 20 | 4·9 | 7·3 | 20 | 10·2 |

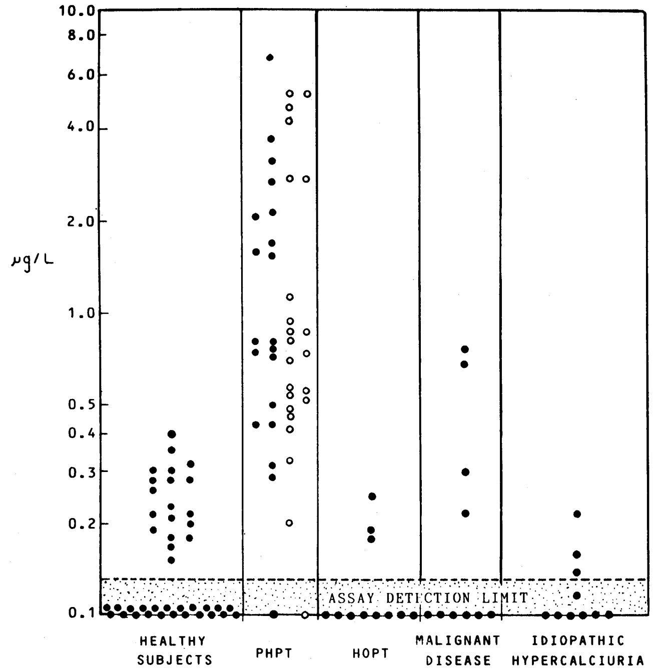

*Figure 2. Serum PTH (C-terminal assay) in healthy subjects and patients with disorders of calcium metabolism. Primary hyperparathyroidism (PHPT) ● proven ○ suspected.*

PHPT is most likely, but on whom parathyroid surgery has not yet been performed. In this group 20 of 23 values were higher than control subjects (see figure 2). Mid-molecule PTH values in PHPT both proven and suspected groups showed values to be raised in 22 of 23 patients.

### Hypoparathyroidism (HOPT)

Eleven patients with hypoparathyroidism (surgical five, idiopathic six) had serum PTH levels assayed by both techniques. The C-terminal method showed that 73% of patients were below the assay detection limit. 54% of healthy subjects also had undetectable PTH levels by this method.

The mid-molecule assay showed serum PTH is undetectable in 73% of parathyroid subjects, but detected PTH in the serum of 92% of healthy subjects.

### Hypercalcaemic malignant disease

Serum PTH values both C-terminal and mid-molecule, measured in patients with hypercalcaemia and malignant disease are shown (see figures 2 and 3 and table 3). Both PTH assays gave some degree of overlap between patients with PHPT and hypercalcaemic malignant disease. This could be reduced when serum PTH was plotted against serum calcium. Mid-molecular serum PTH values showed clearer separation between PHPT and hypercalcaemic malignant patients when data was plotted in this way (see figures 4 and 5).

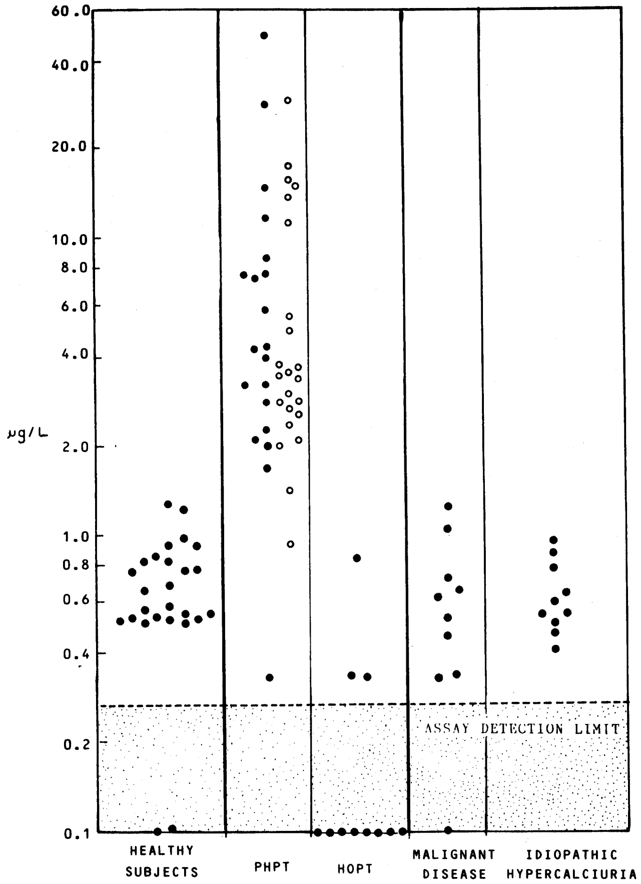

*Figure 3. Serum PTH (mid-molecule assay) in healthy subjects and patients with disorders of calcium metabolism.*

### Idiopathic hypercalciuria (IH)

Serum PTH was measured in the serum of 10 normocalcaemic stone-forming subjects. The C-terminal assay failed to detect PTH in six of 10 patients and four fell within the reference range (see figure 2). PTH values obtained with the mid-molecule assay were not significantly different from control values and serum PTH was detectable in all 10 patients (see figure 3).

*Table 3. Serum calcium and PTH concentration in healthy subjects and patients with disorders of calcium metabolism.*

| Subject | $N$ | Serum total calcium mmol/l | Ionized calcium mmol/l | Serum PTH mid-molecule µg/l | Serum PTH C-terminal µg/l |
|---|---|---|---|---|---|
| Primary hyperparathyroid | | | | | |
| Proven | 20 | 2·99 ± 0·32 | 1·59 ± 0·24 | 4·92 (0·56–43) | 1·01 (0·14–7·43) |
| Suspect | 23 | 2·77 ± 0·18 | 1·49 ± 0·13 | 4·41 (0·75–25·9) | 0·91 (0·12–7·18) |
| Hypoparathyroidism | 11 | 2·01 ± 0·32 | 1·00 ± 0·24 | 0·32 (0·16–0·64) | 0·15 (0·09–0·23) |
| Hypercalcaemic malignant disease | 10 | 3·18 ± 0·35 | 1·75 ± 0·36 | 0·55 (0·20–1·48) | 0·21 (0·05–0·87) |
| Idiopathic hypercalciuria | 10 | 2·32 ± 1·10 | 1·16 ± 0·04 | 0·61 (0·35–1·07) | 0·14 (0·10–0·20) |
| Healthy control | | 2·40 ± 0·07 | 1·20 ± 0·046 | 0·65 (0·31–1·36) | 0·17 (0·08–0·34) |

Serum calcium total and ionized are expressed as: mean SD.
Serum PTH is expressed as: mean (95% range).

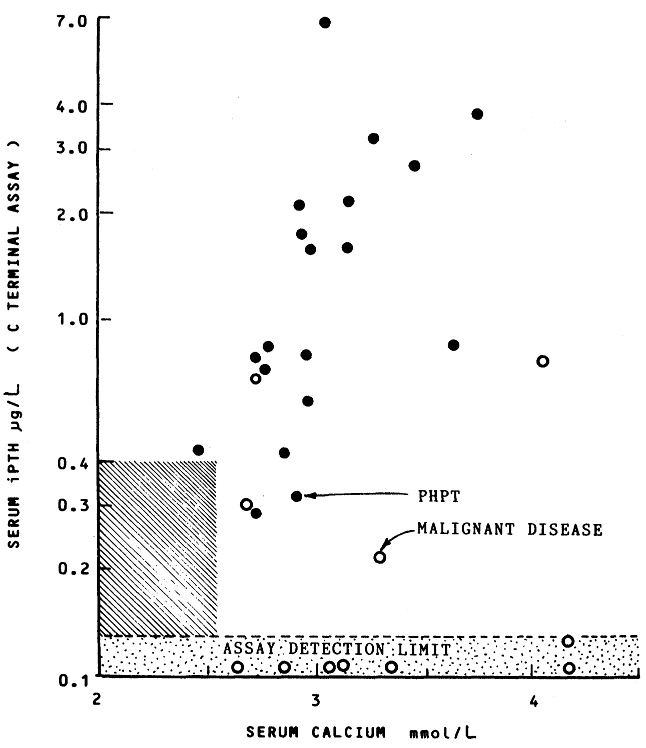

*Figure 4. Serum PTH (C-terminal assay) versus serum calcium in patients with surgically proven primary hyperparathyroidism and malignant disease with hypercalcaemia.*

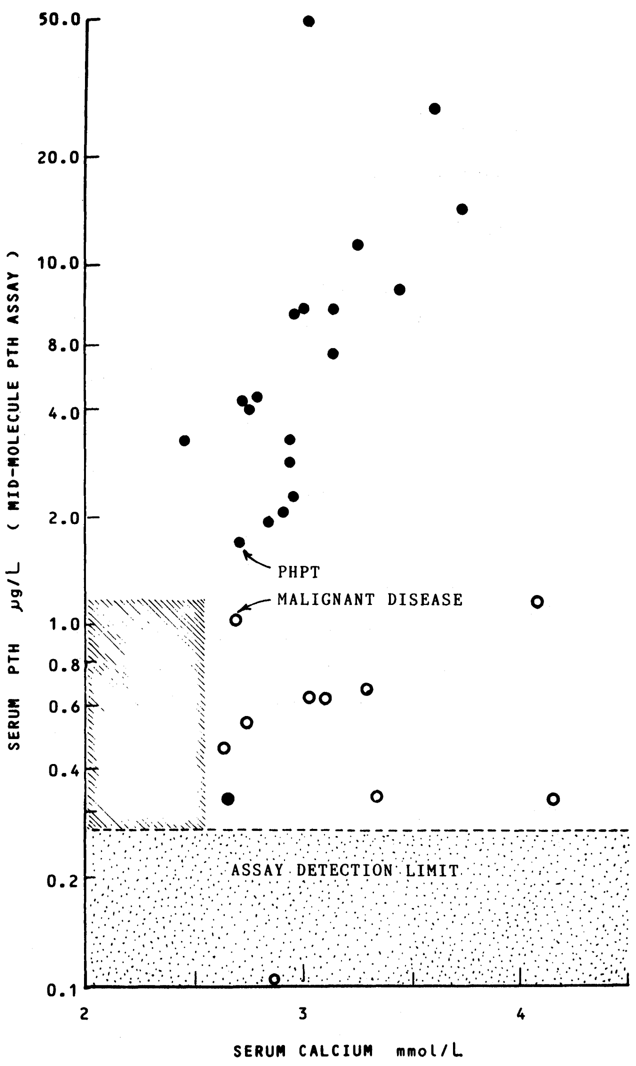

*Figure 5. Serum PTH (mid-molecule) versus serum calcium in patients with surgically proven primary hyperparathyroidism and malignant disease with hypercalcaemia.*

Serum PTH values in all patients assayed by both methods yielded a correlation coefficient $r = 0{\cdot}844$ ($N = 74$). The correlation between PTH values by both methods in the surgically proven PHPT group alone was 0·806 ($N = 20$), and when the proven PHPT and hypoparathyroid groups were combined ($N = 31$) the correlation coefficient was 0·841.

### Discussion

In the past, PTH assays included antisera which recognized either the N-terminal or C-terminal fragments of the parathyroid hormone molecule. PTH assays using an antiserum with mid-molecule specificity are now being more widely used [6–10].

This comparison of C-terminal and mid-molecule PTH assays shows that both commercial assays provide moderately precise methods of determining PTH in serum and both provide results within 24 h. This represents a significant improvement as many of the previously reported in-house assays required four to six days for completion.

Studies reported here and in a previous publication [5] indicate the stability of both mid-molecule and C-terminal fragments of PTH in serum.

The mid-molecule PTH assay was marginally better in the diagnosis of primary hyperparathyroidism and in combination with serum calcium gave better separation between PHPT and hypercalcaemic patients with malignant disease. Nisbet [10], in a recent review of PTH methodologies, also showed mid-molecule to have greater sensitivity in primary hyperparathyroidism than intact PTH assays, serum levels being elevated in eight of nine cases of proven primary hyperparathyroidism. Haver *et al.* [8] evaluating a radio-immunoassay kit measuring mid-molecule PTH fragments analysed 59 patients' specimens, 12 of which were from suspected or proven cases of primary hyperparathyroidism. Serum PTH was raised in 10 of 12 cases giving a sensitivity of 83%. In distinguishing between the hypercalcaemia of malignancy and PHPT, Nisbet found the intact assay to be more successful than the mid-molecule assay.

These studies show the mid-molecule PTH assay to be clearly better than the C-terminal assay in the diagnosis of hypoparathyroidism. Serum PTH values alone can distinguish between patients with hypoparathyroidism and healthy subjects, with one exception. There is considerable overlap between C-terminal PTH values in hypoparathyroid and healthy subjects because the detection limit for PTH is not sufficiently sensitive to detect PTH in 54% of healthy subjects. However, a diagnosis of HOPT can be made when serum calcium is low and PTH undetectable by either method.

Recent reports [11 and 12] have highlighted the increase in hypercalcaemia discovered by biochemical screening. This has led to a considerable increase in the number of patients with asymptomatic PHPT. Rajathurai and Cove-Smith [13] commenting on the prevalence and incidence of PHPT stated that a reliable and reproducible test for PHPT is needed. The report shows that PTH measurement, by either the mid-molecule or the C-terminal assay, provided a useful pointer to primary hyperparathyroidism in the hypercalcaemic patient.

The prevalence of primary hyperparathyroidism varies in the population studied. In the general population it is very low – one report [11] quotes a prevalence of 11 cases per million. Assay of PTH is rarely indicated in this situation. The presence of a clinical sign or symptom, such as renal stone or bone pain, or the finding of a high calcium transfers the patient to a category where the prevalence of primary hyperparathyroidism is much higher. The yield of positive results from PTH assay is therefore much greater when applied in selected populations.

### References

1. Martin, K. J., Kruska, K. A. and Freitag, J. J. *et al., New England Journal of Medicine,* **301** (1979), 1092.
2. Armitage, E. K., *Clinical Chemistry,* **32/3** (1986), 418.
3. Muldowney, F. P., Freaney, R. and McMullin, J. P. *et al., Quarterly Journal of Medicine,* **145** (1976), 75.
4. International Federation of Clinical Chemistry, *Clinica Chimica Acta,* **95** (1979), 163F.
5. Ingle, A. R., Bailey, J. E. and Methews, H. L. *et al., Annals of Clinical Biochemistry,* **23** (1986), 434.
6. Roos, B. A., Lindall, A. W. and Aron, D. C. *et al., Clinical Chemistry,* **53** (1981), 709.
7. Mallette, L. E., Tuma, S. N. and Berger, R. E. *et al., Clinical Chemistry,* **54** (1982), 1017.
8. Haver, V. M., Rinker, A. D. and Ko, B. M. *et al., Clinical Chemistry,* **32**, 11 (1986), 2056.
9. Ivie, W. M., Orwell, E. S. and McClung, M. R. *et al., Clinical Biochemistry,* **19** (1986), 41.
10. Nisbet, J. A., *Annals of Clinical Biochemistry,* **23** (1986), 429.
11. Rao, D. S., *Henry Ford Hospital Medical Journal,* **33** (1985), 194.
12. Harrop, J. S., Bailey, J. E. and Woodhead, J. S., *Journal of Clinical Pathol.,* **35** (1982), 395.
13. Rajatathurai, A. and Cove-Smith, R., *Journal of the Royal Society of Medicine,* **77** (1984), 742.
14. Mundy, G. R., Cove, D. H., Fisken, R., Somers, S. and Heath, D. A. (disputed authorship), *Lancet,* 4i (1980), 1317.

### TRAINING COURSE IN VISCOMETRY

The Department of Trade and Industry's Warren Spring Laboratory is to hold a two-day training course on viscometry on 10 to 11 February 1988. Intended for technicians and research workers who use viscometers in their everyday work, the course will include lectures and hands-on experience on a range of instruments available at the Laboratory. Methods of calculating data results will be included, together with viscometer selection and the testing of difficult materials. The fee is £298.00.

*Further details and a registration form can be obtained from: Ms P. Madhvi, Warren Spring Laboratory, Gunnels Wood Road, Hertfordshire SG1 2BX, UK. Tel.: 0438 741122.*

